# Icaritin: A Novel Natural Candidate for Hematological Malignancies Therapy

**DOI:** 10.1155/2019/4860268

**Published:** 2019-03-28

**Authors:** Xiao-Jing Yang, Ya-Ming Xi, Zi-Jian Li

**Affiliations:** Division of Hematology, The First Hospital of Lanzhou University, Lanzhou, Gansu, China

## Abstract

Hematological malignancies including leukemia and lymphoma can severely impact human health. With the current therapies combined with chemotherapy, stem cell transplantation, radiotherapy, and immunotherapy, the prognosis of hematologic malignancies improved significantly. However, most hematological malignancies are still incurable. Therefore, research for novel treatment options was continuing with the natural product as one source. Icaritin is a compound extracted from a traditional Chinese herb,* Epimedium Genus*, and demonstrated an antitumor effect in various neoplasms including hematological malignancies such as leukemia, lymphoma, and multiple myeloma. In hematological malignancies, icaritin showed multiple cytotoxic effects to induce apoptosis, arrest the cell cycle, inhibit proliferation, promote differentiation, restrict metastasis and infiltration, and suppress the oncogenic virus. The proved underlying mechanisms of the cytotoxic effects of icaritin are different in various cell types of hematological malignancies but associated with the critical cell signal pathway, including PI3K/Akt, JAK/STAT3, and MAPK/ERK/JNK. Although the primary target of icaritin is still unspecified, the existing evidence indicates that icaritin is a potential novel therapeutic agent for neoplasms as with hematological malignancies. Here, in the field of hematology, we reviewed the reported activity of icaritin in hematologic malignancies and the underlying mechanisms and recognized icaritin as a candidate for therapy of hematological malignancies.

## 1. Introduction

Hematological malignancies are known as a series of malignant diseases affecting the blood, bone marrow, lymph, and lymphatic system, which finally lead to dyshematopoiesis. So far, primary treatment includes chemotherapy, radiotherapy, immunotherapy, and autologous or allogeneic stem cell transplantation. Various sorts of anticancer drugs with different mechanisms and targets are developed to attack the cancer cell colony and block disease progression. Outstanding achievements have been made, accompanied by limitations. Conventional drugs bring adverse reactions and sometimes lead to resistance that cannot be ignored [1]. Consequently, new compounds from plants and herbs attract much attention in the hope of devising novel drugs for cancer therapy and prevention [2].

Icaritin, a hydrolytic product of icariin, is extracted from the traditional Chinese herb* Epimedium Genus*. Previous studies demonstrated that icaritin possesses various pharmacological and biological activities in nonneoplastic diseases, including protection of neuron against amyloid-induced neurotoxicity [3], promotion of differentiation from embryonic stem cell into cardiomyocyte [4], enhancement of osteoblastic and suppression of osteoclastic differentiation and activity [5–11], immunomodulation [12, 13], enhancement of self-renewal of mouse embryonic stem cells (mESCs) [14], recovery of UVB-induced photoaging of human keratinocytes [15], and improvement of hematopoietic function in cyclophosphamide-induced myelosuppression mice [16].

Recently, icaritin has attracted great attention in terms of its inhibition of various solid tumors including breast cancer [17–23], hepatocellular carcinoma [24–26], lung cancer [27], oral squamous cell carcinoma [28, 29], endometrial cancer [30, 31], esophageal cancer [32], colorectal cancer [33, 34], glioblastoma [17, 35, 36], ovarian cancer [37], and osteosarcoma [38]. Apart from the common cytotoxic effects, icaritin exhibits some other distinctive properties of restraining tumor progression. For example, icaritin induces cell differentiation, suppresses tumor cell migration, and inhibits cancer stem/progenitor cell growth [18, 32, 39, 40].

Except for investigations of icaritin on the solid tumor, a series of studies on hematological cancer were conducted and proved the significant inhibitory effect of icaritin on various hematological cancer cells, including acute myeloid leukemia (AML), chronic myeloid leukemia (CML), multiple myeloma (MM), and lymphoma [40–44]. Moreover, pivotal signaling pathways were found to be responsible for the proliferative suppression induced by icaritin in hematological cancer also. Though how icaritin suppresses cancer cells through the intricate signal network remains equivocal, these studies indicated that icaritin might be a potential candidate for hematological malignancies. Such review encompasses the proven antitumor effect and mechanism of icaritin as a promising novel agent for hematological malignancies.

## 2. Effects and Mechanisms of Icaritin on Hematological malignancies

### 2.1. Icaritin Induced Apoptosis of Hematological Malignancy Cells

Apoptosis describes a kind of cell death activated by intracellular suicide program. Over the past decades, triggering apoptosis in tumor cells is seemingly regarded as an effective strategy for cancer therapy [20]. Apoptosis occurs through both the extrinsic and the intrinsic pathways. Both pathways are initiated by activation of caspases, and then effector caspases (caspase-3, -6, and -7) are activated to act as executioners of apoptosis [45]. It has been observed that icaritin can induce cell apoptosis in the reported hematological cancer cell lines with flow cytometry analysis [40–44]. Meanwhile, characteristic morphologic changes of apoptosis, like the condensation of nuclear and membrane blebbing, were also found as supporting evidence in MM [27]. Consistently, the proapoptotic proteins, Bax and Bad, were found to be enhanced, while antiapoptotic protein Bcl-2 was suppressed by icaritin in several hematological malignancy cells [41, 42, 44].

Previous findings showed that activated caspase-9, caspase-3, subsequent cleaved PARP, and release of cytochrome c from mitochondria serve as reliable markers of the intrinsic apoptosis pathway [46]. Subsequent studies explored whether the apoptosis induced by icaritin in hematological cancer cells was associated with the activation of the intrinsic pathway and verified that several intracellular signaling pathways in proliferation or apoptosis regulation are involved, such as MEK/ERK, JNK/SAPK, and p38/MAPK [47, 48]. Existing evidence suggested that PI3K/AKT, JAK/STAT, and MAPK/ERK signaling pathways were activated in various hematological malignancies and were considered as a critical target for therapy [49–53]. The pivotal signaling pathways were hence determined to clarify the mechanism of icaritin inducing tumor cell apoptosis. Findings demonstrated that icaritin exerted a remarkable inhibitory effect on the activity of proliferative signaling pathway molecules, restricting the progression of hematological cancer cells. In different hematological cancer cell lines, icaritin was found to work on different signaling pathways; the results were summarized in Table 1.

The JAK-STAT signaling pathway transmits extracellular signals to the nucleus, playing a critical role in transducing signal of a wide array of cytokines and growth factors. These cytokines and growth factors are responsible for various cellular functions, including proliferation, growth, hematopoiesis, and immune response [54–56]. Investigations on hematological malignancies revealed that suppression of icaritin on tumor cells was dependent on inhibition of the JAK-STAT pathway in multiple myeloma [41], chronic myeloid leukemia [40], and extranodal NK/T-cell lymphoma [42].

PI3K-AKT is recognized as a major signaling pathway in cancer development. Through phosphorylation, PI3K-AKT is activated, and the downstream effector participates in regulating tumor cell proliferation, growth, survival, and angiogenesis [57, 58]. Studies suggested that icaritin inhibited the activation of PI3K-AKT, which partly contributed to the inhibition of tumor cell growth in AML and CML [40, 43].

Mitogen-activated protein kinase (MAPK) is a superfamily of protein kinase involved in directing cellular responses to diverse stimuli, such as mitogens, osmotic stress, and proinflammatory cytokines. They play the role of mediating cell functions, including proliferation, differentiation, and apoptosis [59]. Three MAPK families have been precisely characterized, namely, ERK (extracellular signal-regulated kinase), JNK/SAPK (c-Jun N-terminal kinase/stress-activated protein kinase), and p38 kinase [60, 61]. Based on the findings, it has proven that icaritin-regulated expression of MAPK takes part in the suppression of cancer cells in AML and CML [40, 43].

**Figure 1:**
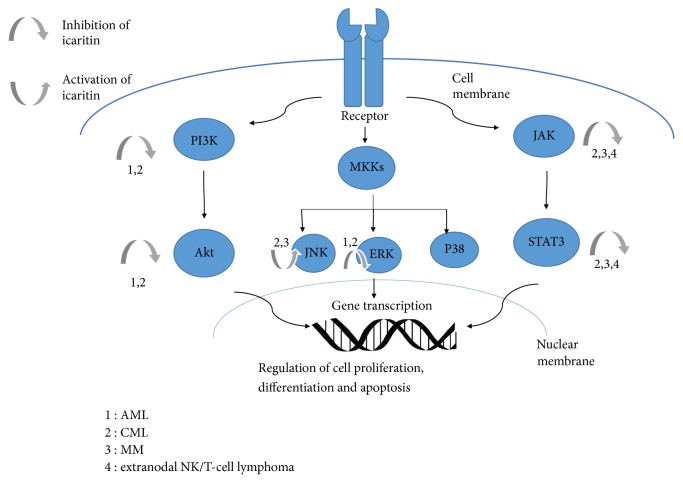
Icaritin induced apoptosis through multiple and differential signaling pathways. Icaritin induces apoptosis by inhibiting PI3K-AKT pathway in AML and CML cells; JAK-STAT pathway in MM, CML, and NKTL cells; and MAPK/ERK pathway in AML and CML cells, while activating MAPK/JNK in CML and MM.

Above all, the mechanism of icaritin inducing of apoptosis through multiple and differential signaling pathways in different hematological malignant tumor cell lines is shown in Figure 1.

### 2.2. Icaritin Arrested Cell Cycle of Hematological Cancer Cells

There are four distinct phases in the cell cycle, namely, G0, G1, S, and G2/M. Cyclins and cyclin-dependent kinases (CDKs) are two crucial groups of molecules in regulating cell cycle progression. In other words, cell proliferation relies intensively on them as they can translate these signals for accurate and active replication and division. Disrupted cell cycle progression might lead to uncontrolled proliferation, accompanied by the occurrence of cancer [62]. Previous investigations proved that icaritin arrested the cell cycle and blocked multiplication of hematological cancer cells. For different cancer cells, icaritin arrested cells at different cell cycle phase. The impact was associated with icaritin acting on the specific cyclins and CDKs. We summarized the results in Table 2.

### 2.3. Icaritin Induced Lytic Replication of Epstein–Barr Virus in NK/T-Cell Lymphoma Cells

Epstein–Barr virus (EBV) is known for its essential role in extranodal NK/T-cell lymphoma (ENKL) etiology [63, 64]. EBV-encoded latent membrane protein 1 (LMP1) participates in the proliferation of ENKL cells by activating several survival signals including MAPK, NF-*κ*B, JAK/STAT, and AKT [65–67]. In the previous study for ENKL, icaritin showed proproliferative and proapoptotic effects likely mediated by inhibiting STAT3 and AKT pathways through downregulating LMP1 [42].

EBV has two distinct lifestyles in an infected human host, including a lytic form, in which the virus lyses the host cells to produce infectious virions, and a latent form, in which the virus reserve the host cells to persist in a dormant state. EBV in ENKL specimens is in a latent form [68]. Recently, various researches suggested that activating the EBV latent-lytic switch could be exploited for therapy of EBV-associated tumors such as BL, nasopharyngeal carcinoma, and gastric carcinoma [69–71]. The lytic-phase genes mainly include the two EBV immediate early (IE) genes BZLF1 and BRLF1 and the early gene BMRF1 [72–74]. BZLF1 encodes the lytic replication activator protein, Zta, which initiates the cascade of BRLF1 and BMF1 to launch the lytic replication [75]. The latent-phase gene EBNA1 is expressed at all latent stages of EBV [76]. Current research demonstrated that icaritin upregulated the expression of BZLF1, BRLF1, and BMRF1 while downregulating the expression of EBNA1 [42]. Besides, treatment with icaritin sensitized the EBV-positive ENKL cells to antiviral ganciclovir (GCV), which was transformed into the active cytotoxic form in the lytic phase [42].

### 2.4. Icaritin Induced Cell Differentiation of Hematological Malignancy Cells

In the survived icaritin treated K562 cells, we found there were morphological changes, such as reduction in cell volume, accompanied with higher hemoglobin level [40]. Flow cytometry analysis showed that the erythrocyte specific antigens, glycophorin A (CD235a) and transferrin receptor (CD71), were increased [77, 78]. All these results made it clear that icaritin can induce K562 to differentiate to the erythroid lineage with substantiated cell toxicity [40].

The p38 has been proved to mediate icaritin induced cardiomyocyte differentiation [79]. In our previous study, icaritin increased p38 phosphorylation and induced K562 cells to differentiate to erythrocyte, while pretreating with inhibitor on phosphorylation of p38 abolished the differentiation induced by icaritin. [40]. Thus, phosphorylated p38 mediates the cell differentiation induced by icaritin in both normal and leukemic cells.

### 2.5. Icaritin Restricted Hematological Cancer Invasion In Vivo

In the investigation with NOD/SCID mice, icaritin dramatically reduced disseminated infiltrations of K562 cells in spleen, bone marrow, and liver without overt bone marrow suppression or weight loss compared to the imatinib-treated group [40]. Additionally, in the NOD/SCID mice subcutaneously inoculated with U266 cells, icaritin led to potent inhibition of tumor growth without apparent body weight loss compared with bortezomib. Immunohistochemistry indicated that icaritin remarkably reduced the expression of p-JAK2, p-STAT3, and VEGF-angiogenesis marker in the transplanted myeloma tissue. Accordingly, icaritin exerted antimyeloma effects in vivo in multiple myeloma [41]. Hence, these findings suggested the specific anticancer effect and relatively less side effects of icaritin for medullary and lymphatic hematological malignancies in vivo.

## 3. Discussion

Currently, icaritin has been proven to possess antitumor activity through extensive mechanisms in several cancer cells, which originated from both solid tumor and hematological cancer. The common cytotoxicity of icaritin in different malignancies includes inhibiting proliferation, inducing apoptosis, and blocking cell cycle [11, 17, 18, 20, 21, 23–29, 31, 32, 35–44, 80–83]. These findings indicated icaritin as a wide-spectrum anticancer agent. The findings of the in vivo test also confirmed the antitumor activity of icaritin in animal disease model and revealed less adverse effect as an advantage compared to conventional chemotherapy agents. The overall properties of icaritin make it a promising candidate agent, which may break through limitations of conventional drugs in the clinic.

For both solid tumor and hematological malignancies, the growth inhibitory effect mentioned above was mediated mainly by the complex cross network of cell signaling pathways including the PI3K/AKT, MAPKs, and JAK/STATs, which ultimately regulated the cell growth, proliferation, and differentiation. Current studies in solid tumors found several molecules with relatively more direct interaction to icaritin above the common signal pathways, such as estrogen receptor in breast cancer cells [11, 23] and sphingosine kinase 1 in hepatocellular carcinoma cells [14]. As presented, icaritin showed inhibition on PI3K/AKT and ERK pathway in both AML and CML cells; suppression on JAK/STAT3 in CML, MM, and ENKL cells; and activation on MAPK/JNK in CML and MM cells (Figure 1). However, the mechanisms of icaritin influencing the signal pathways above are still equivocal.

Moreover, the study of improvement effect of icaritin on hematopoietic function in the cyclophosphamide-induced myelosuppression mice indicated that icaritin could protect chemotherapy-induced bone marrow injury [16]. In other words, icaritin might be an ideal agent which protects normal hematopoietic stem cells and damages hematologic malignancy cells at the same time.

In the context of discovering icaritin, it was also found that it exerts diverse effects on various cancer cells. The primary target of icaritin still needs to be identified by further study. Additionally, the interaction of icaritin and existing antitumor medicine is to be explored to determine whether the synergistic effect or antagonistic effect can be induced.

## Figures and Tables

**Table 1:** Apoptotic activity of icaritin and the underlying signaling pathways in hematological cancer cells.

Cell lines	Pathway of apoptosis	Molecular markers	Signaling pathways	References
Burkitt lymphoma (Raji, P3HR-1)	extrinsic	activated caspase-8,-9 PARP decreased Bcl-2 and c-myc	Inhibition of Bcl-2 and c-myc	[44]

Multiple myeloma (U266)	intrinsic	activated caspase-3,-9, Bak, Bax decreased Bcl-X1	inhibition of IL-6/JAK2/STAT3 Up-regulation of JNK-c-jun	[41]

Extranodal NK/T cell lymphoma (SNK-10, SNT-8)	intrinsic	activated caspase-3,-9, Bax decreased Bcl-2, p-Bad	inhibition of JAK/STAT3 and PI3K/Akt	[42]

Chronic myeloid leukemia (K562)	intrinsic	activated caspase-3,-9 decreased Apaf-1, release of cytochrome C	inhibition of MAPK/ERK and PI3K/AKT up-regulation of JNK/c-jun	[40]

Acute myeloid leukemia (NB4, HL60, U937 Bone marrow mononuclear cell)	intrinsic	activated caspase-3,-7,-9, PARP	inhibition of MAPK/ERK/JNK and JAK2/STAT3 /AKT	[43]

**Table 2:** Cell cycle arrest induced by icaritin in hematological cancer cells.

Cell lines	Arrested phase	References
Burkitt lymphoma (Raji, P3HR-1)	S-Phase	[44]

Multiple Myeloma (U266)	S phase	[41]

Extranodal NK/T cell lymphoma (SNK-10, SNT-8)	G2/M phase	[42]

Chronic myeloid leukemia (K562)	G1 phase	[40]

Acute myeloid leukemia (NB4, HL60, U937 Bone marrow mononuclear cell)	S phase	[43]
